# The pattern of craniofacial encephalocel in Bethel Teaching General Hospital, Addis Ababa, Ethiopia

**DOI:** 10.1186/2045-8118-12-S1-P37

**Published:** 2015-09-18

**Authors:** Tesfaye Mulat

**Affiliations:** 1 Bethel Teaching general Hospital, Ethiopia

## 

Craniofacial encephaloceles are commonly seen birth defects. The incidence is 1 in every 100, 000 live births. Six patients with craniofacial encephaloceles are treated by a combined craniofacial approach. The corrective measure allows reduction of the herniated encephalocele and correction of the craniofacial deformity in the same operation procedure.

All cases admitted from May 2013 until June 13, 2014 to Bethel Teaching General Hospital were identified, and data were collected retrospectively, including demographics, clinical events, MRI and or CT scan findings, surgical techniques, complications and outcomes were analyzed using SPSS.

## Results

See table 1.

**Table 1:** 

Age	Sex	Origin	MRI	Outcome
5	M	Amahara (wollo)	No Hydrocephalus	Good
10	M	Oromiya (Harar)	Mild Hydrocephalus	Good
5	M	Oromiya (Nazerat)	Mild Hydrocephalus	Good
1.5	M	Oromiya (Harar)	No Hydrocephalus	Good
14	M	Oromiya (Harar)	No Hydrocephalus	Good
18	M	Oromiya (harar)	No Hydrocephalus	Good

The MRI finding in four of these patients shows only encephalocele, no hydrocephalus. In two of the cases beside the encephalocel, there is mild hydrocephalus. They need a follow up in six months. Four of the patients weight are below the standard.

All patients were doing well post operatively, no infections, bleeding or other complications.

## Discussions

The surgery performed for all patients were frontal craniotomy including orbital roof was taken. Then encephalocele excision done both from facial and cranial side, followed by correction of the hypertylarism and nasal reconstruction. The surgery takes 5 to 7 hrs. The blood loss was replaces especially for the children below the age of 6. Post operatively they stayed in intensive care unit for two days, thereafter the edema started to decreased, in a week time they were discharged.

Four of the patients were from Oromiya region, the reason is that they were brought by NGO who are working in that region.

## Conclusion

Even though craniofacial encephalocel seems rare in Western countries, it is not rare in this country. One of the reasons could be due to lack of Vit. B during pregnancy. This vitamin is essential for timely closure of the cranial bones. All of these patients are coming from the rural Ethiopia in very low socioeconomic where there is no antenatal care, most of the patients are malnourished. If we provide Vit. B during pregnancy we may reduce the incidence of this pathology.

## Consent to publish

Written informated consent for publication of their clinical details was obtained from the patient/parent/guardian/relative of the patient.
